# Genetic Privacy and Data Protection: A Review of Chinese Direct-to-Consumer Genetic Test Services

**DOI:** 10.3389/fgene.2020.00416

**Published:** 2020-04-28

**Authors:** Li Du, Meng Wang

**Affiliations:** Faculty of Law, University of Macau, Macau, China

**Keywords:** direct-to-consumer genetic testing, Chinese genetic testing providers, genetic information protection, data privacy, consumers’ right to their genetic information, data sharing, data protection law

## Abstract

**Background:**

The existing literature has not examined how Chinese direct-to-consumer (DTC) genetic testing providers navigate the issues of informed consent, privacy, and data protection associated with testing services. This research aims to explore these questions by examining the relevant documents and messages published on websites of the Chinese DTC genetic test providers.

**Methods:**

Using Baidu.com, the most popular Chinese search engine, we compiled the websites of providers who offer genetic testing services and analyzed available documents related to informed consent, the terms of services, and the privacy policy. The analyses were guided by the following inquiries as they applied to each DTC provider: the methods available for purchasing testing products; the methods providers used to obtain informed consent; privacy issues and measures for protecting consumers’ health information; the policy for third-party data sharing; consumers right to their data; and the liabilities in the event of a data breach.

**Results:**

68.7% of providers offer multiple channels for purchasing genetic testing products, and that social media has become a popular platform to promote testing services. Informed consent forms are not available on 94% of providers’ websites and a privacy policy is only offered by 45.8% of DTC genetic testing providers. Thirty-nine providers stated that they used measures to protect consumers’ information, of which, 29 providers have distinguished consumers’ general personal information from their genetic information. In 33.7% of the cases examined, providers stated that with consumers’ explicit permission, they could reuse and share the clients’ information for non-commercial purposes. Twenty-three providers granted consumer rights to their health information, with the most frequently mentioned right being the consumers’ right to decide how their data can be used by providers. Lastly, 21.7% of providers clearly stated their liabilities in the event of a data breach, placing more emphasis on the providers’ exemption from any liability.

**Conclusions:**

Currently, the Chinese DTC genetic testing business is running in a regulatory vacuum, governed by self-regulation. The government should develop a comprehensive legal framework to regulate DTC genetic testing offerings. Regulatory improvements should be made based on periodical reviews of the supervisory strategy to meet the rapid development of the DTC genetic testing industry.

## Introduction

Direct-to-consumer (DTC) genetic testing has gained increasing popularity internationally. The market for DTC genetic testing is estimated to reach 20 billion by 2024 (Global Market Insights, 2018). In recent years, many test providers in China have started to advertise and sell testing products directly to consumers. Similar with providers in the United States such as Ancestry.com and 23andMe, Chinese DTC companies offers genetic testing services for both illness risk determination and lifestyle guidance purposes (Zhao et al., 2013). For example, WeGene, a Shenzhen-based company provides DTC genetic tests for ancestral analysis, personalized sports and weight loss suggestions, nutritional genomics, and genomic medicine, etc. With the increasing influence of popular culture on the public perception of genetics, Chinese consumers’ interest in genetic testing is also estimated to gain a considerable increase in the coming years (Luo et al., 2020). According to a 2018 report developed by Yi Ou, an independent commercial consultant company, the number of consumers for DTC genetic testing will increase exponentially in the next 5 years, from 1.52 million in 2018 to 56.8 million in 2022 (YiOu ZhiKu, 2018).

The scientific community and regulatory authorities have consistently questioned the reliability and clinical validity of DTC genetic testing results as the products have become more widely available to the mass market (Caulfield and McGuire, 2012; Covolo et al., 2015; Webborn et al., 2015). Many studies indicate that the offering of DTC genetic testing may pose risks to privacy and data protection, which may result in potential societal harm to consumers (Hall et al., 2017; Niemiec et al., 2017; Hazel and Slobogin, 2018). Confronted with these controversies and concerns of protecting human genetic resources and biosafety, both the China General Administration of Food and Drug Administration (CFDA) and the State Health Planning Commission (now the State Health Commission) jointly issued the *Notice on Strengthening the Management of Products and Technologies Related to Clinical Use of Gene Sequencing* in February 2014, with the *Notice* suspending all genetic testing services in China (China General Administration of Food and Drug Administration, and State Health Planning Commission, 2014). According to the *Notice*, the technology and products related to clinical genetic testing shall be approved and registered by the CFDA and State Health Planning Commission before entering into the market (Lenore et al., 2016). In July 2014, the CFDA approved the second-generation gene sequencing diagnosis, which can be used for non-invasive prenatal examination for fetal chromosomal aneuploidy disease (Jin et al., 2018). Since then, the CFDA has not approved any other genetic sequencing technology. The *Notice* has played an important role in the clinical application of gene detection technology, but it does not address DTC genetic offerings, thus the supervision of DTC has been operating in an irrefutable gray area.

As a business operator, a DTC genetic testing company should follow the requirements stipulated in the *Law of the People’s Republic of China on the Protection of Consumer* Rights and Interests 2013 (the *Consumer Protection Law*) when collecting and using consumers’ personal information. Companies should inform and obtain the consent of consumers regarding the purposes and scope of collection and use of personal information (The Standing Committee of the National People’s Congress, 2013). The testing company and its employees must keep the consumers’ personal information confidential and should not illegally provide such information to others. To date, there has been no special legislation on DTC genetic testing services. Relevant laws may be applicable to regulate genetic testing offerings, but the main purposes of the current laws are to protect human genetic resources rather than patients’ or consumers’ rights. For example, the National State issued the *Regulation of the People’s Republic of China on Human Genetic Resources Management* (the *Regulation on Human Genetic Resources*) on May 28, 2019 (China State Council, 2019). The new *Regulation* is developed based on a previous National State administrative regulation, the 1998 *Interim Measures for the Management of Human Genetic Resources* (National Intellectual Property Administration, 2019). Compared with the old version, the new *Regulation* places more emphasis on the protection of the privacy and rights of data subjects, including the rights to voluntarily participate and withdraw from the data collection (XinHuaNet, 2019). According to the new *Regulation*, genetic testing providers shall respect consumers’ privacy and cannot collect and use consumers’ genetic data without their informed consent (China State Council, 2019). However, the *Regulation* does not provide detailed requirements for informed consent and privacy protection, as the main goal for the new *Regulation* is to effectively protect and rationally utilize human genetic resources in China. As such, it is focused more on safeguarding public health, national security, and social public interests (China State Council, 2019).

In terms of privacy and personal data protection, China does not have special legislation for the protection of personal data – including genetic data – and privacy at the national law level. In September of 2018, the Standing Committee of the National People’s Congress of China (the SCNPCC) launched a legislative agenda for a comprehensive data protection law, a few months after the European Union General Data Protection Regulation entered into force on May 25, 2018. The plan shows the direction of China’s data protection scheme, and the law is planned to be enacted in 2022 (Feng, 2019). However, relevant laws and standards are applicable to protect consumers’ personal information including genetic data in the context of DTC genetic testing services. For example, the *Cybersecurity Law of the People’s Republic of China* (the *Cybersecurity Law*), which was promulgated by the SCNPCC in 2016, requests that DTC genetic testing providers must not steal or use other illegal means to obtain consumers’ personal information including biometric information, nor illegally sell or provide consumers’ personal information to others (Huang, 2019). Moreover, in 2017, China’s National Information Security Standardization Technical Committee issued the *Personal Information Security Specification*, a national standard that covers the collection, storage, use, transfer, and disclosure of personal information. Personal genetic information is clearly defined as a type of biometric information and categorized as personal sensitive information (China’s National Information Security Standardization Technical Committee, 2017). Different from the *Cybersecurity Law*, which focuses on the regulation of network security and only provides general principles for personal data protection, the *Specification* targeted the protection of personal information and established detailed guidelines for data compliance (Chen and Song, 2018). For example, the *Specification* specifies the details of the content that should be included in the privacy policy and provides a privacy policy template. A genetic testing provider can use the *Specification* as a guideline to set up their specific privacy policy and standards for collecting, storing, using, and processing personal information when dealing with consumer genetic data.

The lack of effective supervision in DTC genetic testing offerings has gained increasing attention from the Chinese news media (Ha, 2019). Many news reports have criticized that the regulatory gap in the industry may result in poor quality testing results and damage to consumer’s health information and privacy (Sui and Sleeboom-Faulkner, 2015). Previous research on US and EU-based DTC genetic testing services indicated that informed consent and privacy protection had been poorly implemented by DTC genetic testing providers. For example, a 2008 study led by Hogarth et al. (2008) highlighted the potential danger of discrimination due to consumer privacy breaches in the implementation of DTC genetic testing. In a 2012 review article, Caulfield and McGuire again recognized the potential privacy issues, revealing rather poor management among DTC genetic companies of addressing consumers’ privacy protection (Caulfield and McGuire, 2012). More recently, in 2018, Overmaat et al. (2018) investigated five leading DTC genetic testing providers in China by ordering products and comparing the different testing results between companies. Their study revealed that, other than technical defects, there were prominent problems in the communication of the test results, with inadequate informed consent being one of the points of concern. However, few studies have been devoted to examining the nuanced perspectives of the Chinese DTC genetic testing offerings. For example, it is unclear what channels providers offered to consumers who are considering the purchase of genetic testing products, as well as what measures the DTC genetic testing companies use to protect consumers’ personal health information, how consumers’ data are shared with a third party, and what rights the consumers have to their data. Aiming to explore these important questions, this study reviews the websites of the Chinese companies and organizations that offer DTC genetic testing services, with a focus on examining all available Terms of Service (ToS), Privacy Policy (PP), and Informed Consent Forms (ICF).

## Materials and Methods

### First Round

From January 17, 2019 to February 27, 2019, we used Baidu.com, the most popular search engine in China, and search keywords: “genetic testing” (in Chinese: “
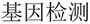
”) to identify and collect providers that use the DTC model to market genetic testing services and products. Based on this search, we collected 90 DTC genetic testing offerings. We then visited the websites and captured the webpages of the providers and downloaded all available documents related to the terms of service, privacy protection, and informed consent. It is essential to clarify that our study focuses on examining organizations that mainly offer genetic testing services and products.

From May 7, 2019 to June 18, 2019, one of our authors analyzed the websites using a coding framework focused on the following perspectives: (1) channels provided for purchasing genetic testing products and services; (2) informed consent; (3) privacy issues; (4) strategies used to protect consumers’ personal health information; (5) data sharing with a third party; (6) consumers’ rights to their health information; (7) responsibility for data breach; and (8) specific laws or legal protections mentioned. These eight perspectives were established based on previous studies on legal and ethical issues associated with DTC genetic testing and personal data protection in the big data era in healthcare (Hogarth et al., 2008; Mostert et al., 2016). The coding framework included 20 items which were developed based on an exploratory content analysis of 30% of the dataset. In this round of content analysis, we found that three websites that were initially identified as genetic testing providers were no longer accessible, and thus were removed from our dataset.

### Second Round

Later, on July 1, 2019, a new Decree of the State Council of China took effect, the *Regulations on the Management of Human Genetic Resources of the People’s Republic of China*. Since the collection of website information and the content analysis of consumer-related legal documents were carried out before the implementation of the new *Regulation*, we took advantage of the opportunity to examine how Chinese DTC genetic testing providers reacted to the new *Regulation*. For example, we attempted to identify if there were updates to their ToS, PP, and ICF to meet the requirements of the updated requirements 3 months after the *Regulation* took effect. Consequently, we revisited the websites of the companies collected in our dataset and again reviewed the content of the websites and the legal documents to determine if any changes were made to comply with the new *Regulation*. In this second round of collection and analysis, we found that the original links of nine websites were broken. Five of these nine problematic websites changed to new domain names, and four of the nine websites were completely invalid. As a result, our dataset in the second round consists of 83 accessible websites. We analyzed these 83 websites using the same coding framework from September 2, 2019 to September 20, 2019. We used the 83 websites as the final dataset for this research and compared the results of the two rounds of analyses.

For both rounds of content analysis, 30% of the websites were randomly selected to compare the consistency of coding results in order to verify the reliability of the coding. After obtaining the 30% of websites, an independent coder searched the URLs of the websites, reviewed the content and available privacy policy, informed consent forms, and the terms of service. We calculated the agreement between the two codes, using Cohen’s Kappa evaluation. The agreement was between 0.85 and 1.00 for all coding frame items, which indicates substantial to perfect agreement (Landis and Koch, 1977).

## Results

### Methods Provided for Purchasing Genetic Testing Services and Products

Fifty-seven providers of DTC genetic testing services and products offered online purchase options via their websites. Twenty-six providers only accepted traditional banking transfers as payments after counseling with consultants via telephone. For providers that offered online purchases, 21 providers required consumers to register on the websites before they could order services and products online, and five websites integrated with other e-commerce platforms^1^,^2^, which allowed consumers to order genetic testing products or services through a third party. In particular, we found that WeChat, the most widely used social media platform in China, became a popular vehicle for providers to promote their products and services. Thirty-two providers used WeChat to introduce their products and to offer follow-up services to consumers. Consumers could order products and make payments via providers’ WeChat stores or by transferring money after adding the providers as WeChat friends. In contrast, very few providers (*n* = 3) used QQ, an instant messenger that at one time was the most widely used in China, as a promotion platform. The case for the relatively few providers using QQ to reach consumers might arguably be due to the increase in WeChat users.

### Informed Consent

We only identified 6% of providers (*n* = 5) that provided informed consent forms on their websites. The promulgation of the new *Regulation* did not make a big difference in the availability of informed consent forms. Only one provider added an informed consent form to its website after the new *Regulation* took effect.

Every informed consent form exceeded 500 words. Two informed consent forms were between 1,000 and 2,000 words, and two had more than 2,000 words. In terms of content, all informed consent forms mentioned the protection of consumer privacy and the risk of information leakage. One provider enumerated all possible risks of implementing genetic testing, including: (1) consumers or their families may feel uncomfortable because of survey questions or genetic data results; (2) information leakage due to security breach; (3) information leakage caused by consumers sharing accounts and passwords with others; and (4) other currently unforeseeable risks. Three providers mentioned that their genetic testing reports were predictive, and which can only be used as a health consulting reference not as a clinical diagnostic basis.

With regard to the remaining websites (*n* = 78), although they failed in providing specific informed consent forms, 13 providers did mention the informed consent procedure on their websites. For example, 11 websites indicated that informed consent procedures would be implemented by providers during or after the purchases of the genetic testing services and products.

### Privacy Issues

In our first-round examination, we found that 38 websites had provided accessible links to privacy policies. During the second round of collection, one company within the 38 was removed from the dataset because its website was no longer accessible.

Consequently, we identified 37 websites in total that offered a privacy policy. This means, however, that more than half of the websites (55.4%) did not provide consumers with a privacy policy (*n* = 46). Four companies mentioned privacy issues, but they did not offer a privacy policy on their websites. For example, two websites stated in their FAQ section that they had addressed privacy issues in their privacy policies, but offered no links to the privacy policies on their websites. Similarly, two websites had privacy policy tags at the bottom of the webpages, but clicking on these tags did not lead to valid links to the privacy policies. Moreover, in our second-round investigation, we did not identify an obvious difference in the provision of the privacy policy. In fact, except for one provider who updated the privacy policy by adding one sentence addressing the liability distributions in the event of a privacy breach, the rest of the providers did not make any changes to their privacy policies.

Privacy policies with less than 1,000 words were generally not written in an agreement format, instead functioning more like privacy statements, where short sentences were used to indicate service providers’ attempts for protecting the consumer’s privacy (see Table 1). In contrast, privacy policies with 1,000 to 2,000 words or more were generally written in an agreement format, including definitions of the terms involved in the agreements, detailed explanations of the rights and obligations of users and providers, and applicable laws. However, only privacy policies with more than 2,000 words meet the requirements of the *Personal Information Security Specification* on the content of privacy policy.

**TABLE 1:** Examples of different lengthy privacy policy used by DTC genetic testing companies.

**Word counts**	**Number of providers (number of providers whose privacy policy covers the content required by the *Personal Information Security Specification*)**	**Summary of the main content**
Less than 200 words	9 (0)	Service providers will protect the genetic privacy of consumers, but no specific protection measures are mentioned.
200–500 words	12 (0)	Privacy clauses focus on privacy protection in the collection and process of personal data, the use and disclosure of personal data, and privacy security. However, the specific contents and measures are not mentioned and explained.
500 – 100 words	4 (0)	Privacy clauses clarify the rights and obligations of users and providers. The exemption clause is included.
1,000–2,000 words	7 (0)	The privacy policy is in the format of an agreement. In general, it includes: definitions of terms used in the agreement, the rights and obligations of users and providers, and applicable and governed laws.
More than 2,000 words	5 (5)	The privacy policy is comprehensive and meets all the requirements of the *Personal Information Security Specification* on the content of privacy policy, including the collection and use of users’ personal information, the use of cookies and similar technologies, the sharing, transfer, and disclosure of users’ personal information, the measures for protecting user’s personal information, the rights of users, and the methods for dealing with children’s information, etc.

### Strategies Used to Protect Clients’ Personal Health Information

**FIGURE 1:**
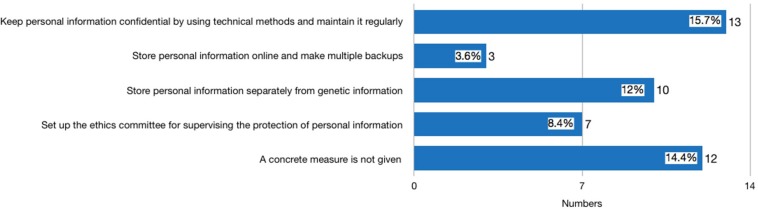
What measures will the provider adopt to protect general personal information.

**FIGURE 2:**
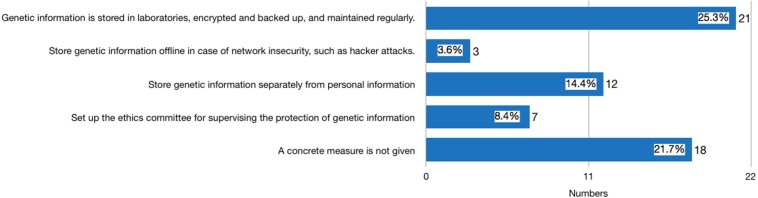
What measures will the provider adopt to protect genetic data.

Thirty-nine websites published statements either on the front page of their website, their ToS, PP, or ICF declaring that they use measures to safeguard the security of consumers’ genetic information. Among these 39 providers, three were identified in the second round of investigation after the new *Regulation* was issued. It is worth noting that 29 privacy policies had distinguished general personal information (GPI) of consumers, such as website registration ID, social security ID, and health information, from their genetic information. Seventeen providers proposed concrete measures for protecting consumer GPI (see Figure 1). The most frequently mentioned measures include: using technical methods to keep the confidentiality of GPI and maintain it regularly (mentioned by 13 providers); storing consumer GPI separately so that staff analyzing the genetic information are not able to identify the subject of the genetic data (mentioned by 10 providers); establishing an ethics committee for supervising the protection of GPI (mentioned by seven providers). In terms of consumer genetic data, the measure that discloses how consumer genetic information is stored in laboratories, encrypted, backed up, and maintained regularly is the most frequently stated method (mentioned by 21 providers) (see Figure 2). However, none of the providers had clearly stated how long the consumers’ data would be kept, and whether the data will be eventually destroyed.

### Data Sharing With a Third Party

While 62.7% of providers (*n* = 52) did not address the reuse, selling, or sharing of information gathered from consumers in their informed consent forms, privacy policies, or terms of service, 28 providers stated that with additional client permissions, the providers can reuse and share the clients’ information for non-commercial purposes. Two providers mentioned that they would not sell a client’s information unless having obtained the client’s additional permission. Only one company mentioned that it would reuse and share a client’s information for non-commercial purposes without the client’s further permission. Specifically, the company stated that: in a case where the third party agrees to assume the same responsibility of protecting users’ privacy as the company does, the company can provide the third party with users’ registration and other information without consumers’ further permissions. Moreover, 22 websites mentioned the compelled disclosure of personal health information in accordance with laws in either the ICF or PP. In regards to information disclosure, 73.5% of providers (*n* = 61) did not make any statements about mandatory information disclosure on their websites.

### Consumer Rights to Their Health Information

Twenty-three providers granted consumer rights to their health information, while the rest of the providers (*n* = 60) kept silent in this respect. In general, three types of rights have been granted by the DTC genetic testing providers to their customers. Among these, the most frequently mentioned right is the consumers’ right to decide whether providers can use their genetic data for follow-up research or provide their data to third parties (mentioned by 21 providers). Nine providers stated that consumers have the right to view and change their personal data or to remove their data from the providers’ database. Only one company mentioned that consumers have the right to be informed of follow-up use of their data, which includes: (1) using users’ genetic data to develop new products based on gene sequencing results; and (2) using genetic data for the latest interpretation of existing projects, interpreting the latest scientific literature, and recalculating existing projects more accurately.

### Accountability and Responsibility for Data Breach

While nearly 70% of providers (*n* = 58) did not inform consumers of the risk of accidental information leakage, 30.1% of providers (*n* = 25) mentioned relevant risks on their websites, e.g., hacker attacks, internet errors, and other unforeseeable accidents. Among these 25 providers, all stated that the company would strive to protect customers’ privacy, preventing their health information from being disclosed arbitrarily. However, in terms of the distribution of responsibility for the breach of privacy, fewer providers (*n* = 18) made explicit statements regarding whether they shall bear liability. In particular, nine providers did not address who would assume responsibility in the event of a data breach, but clearly stated that providers will be exempted from liability if the leaks are due to causes beyond their control. Eight companies specified only the consumers’ responsibilities but not the obligations of providers in a breach. Just one company stated in their PP that in the event of a data leakage, that the liability would fall on the source of the breach, i.e., the source of the leak will bear the responsibility.

### Law Mentioned

Twenty-three Chinese DTC genetic testing websites in their ICF, ToS, or PP stated that the company would comply with relevant Chinese laws in collecting, storing, and using consumer genetic data during and after genetic testing services. However, among these 23 websites, only four companies mentioned concrete laws, e.g., the *Cybersecurity Act* (mentioned by two companies), the *Regulation on Human Genetic Resources* (mentioned by one company), and the *Interim Measures for the Management of Human Genetic Resources* (mentioned by one company), which had been replaced by the *Regulation on Human Genetic Resources*.

It is worth noting that three companies mentioned the *Health Insurance Portability and Accountability Act* (HIPAA), the United States legislation that protects the privacy and security of Americans’ medical information in the PP, ICF, or data protection agreement for dealing with issues of privacy protection. We found that HIPAA was applied in different ways. For example, one company mentioned that the storage of consumers’ genetic data will strictly abide by the HIPAA requirements. One company stated that for international users, the process of the genetic testing services will follow HIPPA standards. Another company used the HIPAA as a reference for their practice of storing consumers’ genetic data. In addition, one company promised that it would abide by the *Hong Kong Personal Data (Privacy) Ordinance* to carry out genetic testing services.

## Discussion

Our research indicates that Chinese DTC genetic testing companies have begun to take action in protecting consumer genomic data privacy. For example, many providers developed specific measures for protecting the security and privacy of consumer health information. Our research particularly indicates that some DTC genetic testing providers separated the consumer general health information from their genetic information and used different measures to protect the two types of data. This may reflect the industry’s growing awareness of the sensitivity of genetic information and the need for using special measures to protect consumers’ genetic data. Moreover, consumers’ rights to their health information were recognized by many DTC genetic testing companies. For example, consumers have the right to access their personal data or have their information removed from company records. Nonetheless, our research has identified several legal concerns that Chinese regulatory bodies should immediately address.

First and foremost, we found that the provision of informed consent forms is not a common practice for the Chinese DTC genetic testing providers. This finding is in agreement with previous studies on international DTC genetic testing companies, where informed consent practices were found to be inadequate and sometimes misleading (Howard et al., 2010; Lachance et al., 2010; Niemiec et al., 2016). Until very recently, informed consent in China was not thoroughly implemented in general clinical practice (Wu et al., 2019). Previous studies have indicated that informed consent has grown in importance over the years as an effective strategy in softening the increased tensions between physicians and patients (Bal and Brenner, 2015). Still, consumers may not be aware of the importance of informed consent in DTC genetic testing services. For instance, many consumers may not realize that they need a supportive process to fully and properly understand the purposes and possible results of the testing, and, more importantly, the impact of testing results and genetic information on their health and other interests (Deng and Liu, 2017). In particular, as opposed to other countries where informed consent is a legal requirement for implementing genetic testing services (Knoppers et al., 2015), China has not established such legal requirements for requesting a mandatory informed consent process before receiving DTC genetic testing.

Moreover, we found that many Chinese DTC genetic testing companies offer both non-health-related tests and tests for health purposes. This raises further concerns about whether the same rules should be applied for regulating informed consent in both types of gene sequencing applications. In countries where regulatory measures for DTC genetic testing services are comparatively loose, they generally have strict requirements of informed consent for health-related genetic testing. For example, in the United States, the Presidential Commission for the Study of Bioethical Issues stated that if the genetic testing is prescribed due to clinical purposes, physicians have to present informed consent to patients due to their fiduciary duties to patients (Niemiec et al., 2016). In China, although the new *Regulation on Human Genetic Resources* specifies that informed consent should be obtained from providers of human genetic resources before collecting and using their genetic information, the purposes of the *Regulation* are mainly based on the concerns of safeguarding public health, national security, and social public interests, rather than patients or consumers’ health rights and interests in the context of genetic testing. Given the lack of regulation in China that can guide and monitor informed consent procedures for both clinical and non-health-related genetic testing, consumer rights for health and information are left without adequate protection.

Compared with the number of informed consent forms provided, more Chinese DTC genetic testing companies have addressed issues related to consumer privacy protection. For example, 37 websites offered a link to a privacy policy. This finding is consistent with existing research that privacy concerns have been increasingly addressed by international DTC genetic testing providers (Webborn et al., 2015). That being said, in the Chinese DTC genetic testing market, more than half of the websites we analyzed did not offer a legal statement on consumer privacy protection. With regards to the privacy policies offered by the DTC genetic testing providers, the majority were short and incomplete, which did not cover the content required by the *Personal Information Security Specification* on privacy policy. In particular, 3 months after the *Regulation on Human Genetic Resources* took effect, we did not identify a significant change in the DTC genetic testing industry for improving the practice of informed consent and consumer privacy to comply with the new legal requirements. Only one genetic testing provider had given an update to its informed consent form on its website, in that they offered a link to an informed consent form and added one short sentence for the protection of consumers’ privacy in the form. This illustrates that, without an established comprehensive personal data protection law, both the *Cybersecurity Law* and the regulatory measure on genetic resources management had very little influence on promoting and advancing the protection of privacy and implementation of informed consent in DTC genetic testing services.

Although several relevant laws, such as the *Consumer Protection Law*, *Cybersecurity Law*, and the *Regulation on Human Genetic Resources* are applicable to regulating the genetic testing market, there is no special regulatory regime covering important issues associated with the implementation of DTC genetic testing services, e.g., informed consent, privacy protection, and transparency about how consumer genetic data is used, collected, and shared. Thus, the current Chinese DTC genetic testing market operates as a self-regulated mechanism. Moreover, “soft laws,” such as best practices and a code of conduct, are also missing in the regulations of DTC genetic testing. We did not identify any established voluntary best practices guidelines for genetic testing services (Park et al., 2019). BGI, the biggest player in the Chinese gene sequencing market, leveraged its role in the field by organizing a focused group meeting with other genetic testing companies for the purpose of developing a group standard for genetic testing reports. As an outcome of the discussion, the *Specification for Gene Detection and Reporting of Clinical Monogenous Hereditary Diseases* was issued in 2018, becoming the first practical standard in the genetic testing industry (Hui et al., 2018). However, as BGI stated in the article that every process of the genetic testing service requires corresponding standards, and the *Specification* for clinical genetic testing report alone is far from adequate (Hui et al., 2018).

Without a sufficient and effective regulatory framework for DTC genetic testing services, consumers may face increased risks of losing control of their genetic information and privacy breaches. Specifically, we found that many companies failed to provide meaningful information to consumers concerning the security of genetic data and how the data will be used with a third party. For example, some companies granted consumers the right to authorize the use of their data, but if thorough informed consent procedures are not provided, consumers are unlikely to know for what purposes their genetic data will be used and how their data will be handled by a third party (Tomlinson et al., 2016). This is especially problematic if there is no definition of “third party,” as well as details regarding how the genetic testing company will safeguard privacy when data is transferred to a third party. As a result, the risk of consumer data leakage is very high. Moreover, many companies did not state clearly what their liability would be in the event of a data security breach. In general, the testing companies kept silent in regards to their responsibilities in the event of a privacy breach, though some were explicit in releasing themselves from any liability in cases when breach incidents were caused by events out of their control (Hazel and Slobogin, 2018). Given these concerns, consumers should be careful and diligent when choosing genetic testing services and products (Badalato et al., 2017).

Our research indicates that the United States *HIPPA* legislation was mentioned by several Chinese DTC providers. These companies highlighted their efforts to protect consumer privacy and data security by strictly complying with the legal requirements detailed in *HIPPA*. In terms of anti-genetic discrimination in the health insurance realm, the China Insurance Regulatory Commission (the former entity of the China Banking and Insurance Regulatory Commission) demonstrated the restrictions on the use of genetic testing results in health insurance in the *Measures for the Management of Health Insurance (draft for comments)* – a 2017 version for the update of *Measures for the Management of* Health Insurance 2006 (China Insurance Regulatory Commission, 2017). Articles 11 and 16 of the 2017 *Measures* require that insurance companies protect the privacy and confidentiality of policy holders, the insured, and the beneficiaries, and that insurance companies should not add premiums to policy holders based on their genetic testing data and genetic information, other than the family genetic history of a disease. Moreover, according to Article 36 of the 2017 *Measures*, insurance companies should not require policy holders or the insured to take genetic tests, and that the genetic testing results are prohibited from being used as a condition for the verification of insurance. As the 2017 *Measures* have not been passed and entered into effect, further research is needed to examine and review the impact of the new regulations on the protection of private health information within the context of DTC genetic testing.

In addition, our research indicates that social media has played an increasingly important role in promoting genetic testing services and products. On the one hand, social media, as previous studies indicated, could be a useful tool to increase patients’ knowledge of genetic testing and risk assessment for certain types of cancers (Attai et al., 2015). A recent investigation by Roberts et al. (2019) on the public reactions on Twitter to the government’s authorization of DTC genetic testing for BRCA1/2 variants associated with breast cancer corroborated the substantial impacts of social media in this regard. Their research revealed the potentials of social media to become the main platform for disseminating and exchanging information about genetic research and technology, as well as a powerful medium for consumer testimonials (Roberts et al., 2019). In this regard, social media platforms could be used to raise public awareness of the inadequacies of privacy measures taken by gene testing providers. On the other hand, a large number of studies on social media’s role in promoting new technology has indicated that information about new biotechnology shared through social medial was usually unbalanced and misleading (Galata et al., 2014). Although we did not analyze what content had been promoted on WeChat platforms about genetic testing, we suggest regulatory agencies focus their attention on the legal and ethical issues associated with using social media for the promotion of genetic testing services and products. Given these concerns, further studies on the role of social media in DTC genetic testing services and products are needed.

## Conclusion

Our study indicates that DTC genetic testing has become an emerging market in China. Eighty-three Chinese companies were identified as promoting genetic testing products directly to consumers. The existing applicable regulations on genetic testing are mainly focused on human genetic resource security and protection, and no special legislation has been developed to regulate DTC genetic testing offerings. Without an established legal regime, the availability for informed consent forms and policies for consumer data and privacy protection within the industry are self-imposed by DTC genetic testing companies. Moreover, the industry has not established any best practices guidelines for implementing DTC genetic testing services. As a result, the current DTC genetic testing business is running in a regulatory vacuum and is governed by a self-regulation mechanism. Our study indicates that the limits of this self-regulation model is obvious. Informed consent forms were generally not provided by DTC genetic testing companies, and a privacy policy was only available on less than half of all providers’ websites we examined. For the majority of DTC genetic testing companies, consumers’ autonomy for purchasing genetic testing is unable to be guaranteed, and there is a lack of transparency about how consumer genetic information is used and shared. As a result, consumers are left without adequate protection. Their genetic information might be illegally used or shared to a third party without their permission. We, therefore, urge that adequate and effective regulatory oversight over DTC genetic testing offerings should be developed. In particular, a clear and sufficient informed consent form and privacy policy should be provided on all DTC genetic testing providers’ websites (Hendricks-Sturrup and Lu, 2019). Moreover, to meet the increased requirements of data protection and the demands of data sharing, regulations should be developed to render a legitimized systematic approach to the collection, use, and sharing of consumer genetic databases.

As the industry keeps evolving, some challenging issues associated with the provision of DTC genetic testing require further studies. For example, social media has been frequently used by DTC genetic testing companies as an alternative way to promote genetic testing services. The involvement of social media could bring opportunities for raising public awareness on potential privacy risks with DTC genetic testing. It may also trigger regulatory challenges in supervising the dissemination of truthful and balanced information on genetic testing via social media platforms. Additionally, some DTC genetic testing companies have distinguished consumers’ general health information from genetic information and used different methods to safeguard data security. This raises the question of whether different information protection rules should be developed and applied to different types of consumer health data. Given all these potential challenges and the growing industry, the development of regulations on genetic testing call for interdisciplinary perspectives, and it is essential to examine periodically the regulatory framework on genetic testing services.

## Data Availability Statement

The datasets generated for this study are available on request to the corresponding author.

## Author Contributions

LD designed the study. MW collected and analyzed the data. LD drafted the manuscript. MW made revisions to the manuscript.

## Conflict of Interest

The authors declare that the research was conducted in the absence of any commercial or financial relationships that could be construed as a potential conflict of interest.

## Footnotes

1 JD.com

2 Taobao.com
