# Supplementary material for: Genetic Privacy and Data Protection: A Review of Chinese Direct-to-Consumer Genetic Test Services
Source: Front Genet. 2020 Apr 28;11:416. doi: 10.3389/fgene.2020.00416 (PMC7205185; doi:10.3389/fgene.2020.00416)
Supplement: Supplementary file 1 [file Data_Sheet_1.PDF]

Coding book for genetic privacy and data protection: A review of Chinese direct-to-consumer genetic test services

1. How can consumers order genetic testing services?
A. via Website
B. via an E-commerce website
C. via WeChat (QR code)
D. via QQ
E. via Telephone

2. Does the website provide a link to an informed consent form?
A. Yes, and the link is easy to find ("easy": no need to go through additional webpages; no need to scroll down; using a clear font, simple background, and a reasonable text size)
B. Yes, but the link is hidden or otherwise difficult to locate
C. No, not at all.

3. If an informed consent form is provided, what is the word count?
A. 200 words or less
B. 200 words ~ 500 words
C. 500 words ~ 1000 words
D. 1000 words ~ 2000 words
E. 2000 words or more

4. If an informed consent form is not provided on the website, does the website offer information about the informed consent procedure?
A. Yes
B. No

5. If yes, how does the website describe the informed consent procedure?
A. Informed consent will be implemented by the service provider when buying the service.
B. No clear description was given

6. Does the website provide a link to their privacy policy?
A. Yes, and the link is easy to find ("easy": no need to go through additional webpages; no need to scroll down; using a clear font, simple background, and a reasonable text size)
B. Yes, but the link is hidden or otherwise difficult to locate
C. No, not at all

7. If a privacy policy is provided, what is the word count?
A. 200 words or less

B. 200 words ~ 500 words
C. 500 words ~ 1000 words
D. 1000 words ~ 2000 words
E. 2000 words or more

8. If a privacy policy is provided, does the policy meet the requirements of the *Personal Information Security Specification* on the content of the privacy policy?
A. Yes
B. No

9. If a privacy policy is provided, does it distinguish general personal information (e.g., website registration ID, personal ID, health information, etc.) from genetic information?
A. Yes
B. No

10. If the website distinguished general personal information from genetic information, how does the website describe the measures that the provider will adopt to protect such information?
A. Provider will keep personal information confidential by using technical methods and maintain it regularly.
B. Provider will store personal information online and make multiple backups.
C. Provider will store personal information separately from genetic information.
D. Provider will set up an ethics committee for supervising the protection of personal information.
E. No concrete measure were given.

11. Does the website mention that the provider will adopt measures to protect the clients' genetic information?
A. Yes
B. No

12. If yes, what measures are mentioned?
A. Genetic information is stored in laboratories for a long time, encrypted and backed up, and maintained regularly.
B. Provider will store genetic information offline in case of network insecurity, such as hacker attacks.
C. Provider will store genetic information separately from personal information and manage the de-identification of genetic data.
D. Provider will create an ethics committee for supervising the protection of genetic information.
E. No concrete measure were given

13. If the provider does offer a link for an informed consent form/privacy policy, does the informed consent form/privacy policy include articles about the company's right to reuse/sell/share clients' information with other entities?

A. Yes. Under a client’s further permission, the company can reuse/share the client’s information for non-commercial purposes.
B. Yes. The company can reuse/share a client’s information for non-commercial purposes without the client’s further permission.
C. Yes. The company cannot sell a client’s information unless the client’s further permission is obtained.
D. Yes. The company will not sell or rent personally identifiable information to any other company or organization for direct marketing purposes. The company may reveal information about you to unaffiliated third parties.
E. Yes. The company can reuse/share/sell the identifiable health information without clients’ further permission.
F. No. The company does not include such articles.

14. Does the informed consent form or privacy policy (terms) mention the compelled disclosure of personal health information?
A. Yes
B. No

15. Does the informed consent form or privacy policy (terms) mentioned about consumers’ rights to their data?
A. Yes
B. No

16. If yes, what rights were mentioned?
A. Consumers have the right to decide whether the websites could use their data for follow-up research or to provide their data to third parties.
B. Consumers have the right to view and change their own data or have the right to ask websites to remove their data.
C. Consumers have the right to know that the websites are using their data.

17. Does the informed consent form or privacy policy (terms) mention the risks associated with data breaches?
A. Yes
B. No

18. According to the policy, if a breach of consumers’ data occurs, who is the responsible party?
A. The consumer
B. The company
C. N/A

19. Does the informed consent form or privacy policy (terms) mention the Chinese data protection law?
A. Yes
B. No

20. If yes, what laws does it mention?
A. Cybersecurity Act (《网络安全法》)
B. Civil law
C. Tort Act
D. Regulation on Human Genetic Resources (《人类遗传资源管理暂行办法》)
E. The Personal Data (Privacy) Ordinance Hong Kong (《个人资料（隐私）条例》)
F. No specific law is given
