# Supplementary material for: Genetic Privacy and Data Protection: A Review of Chinese Direct-to-Consumer Genetic Test Services
Source: Front Genet. 2020 Apr 28;11:416. doi: 10.3389/fgene.2020.00416 (PMC7205185; doi:10.3389/fgene.2020.00416)
Supplement: Supplementary file 2 [file Data_Sheet_2.PDF]

Chinese DTC Genetic Testing Websites

Order	Name of provider	URL
1	水母基因	https://www.somur.com/consumer
2	易毕恩基因公司	http://www.epican.cn/product/
3	佳学基因	http://www.jiaxuejiyin.com
4	爱基因	http://www.aijiyin.com.cn/?mod=about&id=12
5	检爱网	http://www.dnalover.com
6	泛生子	http://www.genetronhealth.com/index.html
7	博奥检验	http://www.medlab.cn/article.php?id=102
8	首度基因	http://www.shoudujiyin.com
9	吉因宝	http://www.jiyinbao1.com/index.php
10	各色 DNA	https://www.gesedna.com/privacy-policy/
11	久久基因	http://www.99jiyin.com/help.html#ysbh
12	艾尔基因检测中心	http://www.aiergene.com/nd.jsp?id=23#_np=103_632
13	SkinDNA	http://www.skindna.vip/international/news.php?lang=cn&class2=199
14	盛景基因	https://www.sjjiyin.com/about/help/_class/class3
15	达安基因	http://daan-gene.com/index.php
16	赢通医检	http://www.krhmc.com/nd.jsp?id=168#_np=2_356
17	联川生物	http://www.lc-bio.com/health.html
18	360°健康	http://www.360jiyin.com/index.php/Article/aboutUsList?id=2
19	天使基因	http://www.angelgene.cn/theme/2.html
20	利普康	http://www.repconex.com/index.php/home/index/shop
21	玩因的	https://www.genefun.net/page/test/

22	微基因	https://www.wegene.com/help/#category=4
23	星云基因	https://www.nebulagene.com/node/111
24	上海赛安基因城	http://www.c-genecity.com/about/2.html
25	壹基因	http://www.1gene.com.cn/health.html
26	23GENEBANK	http://www.23genebank.com/fuwu/shengming
27	拓普基因	http://www.topgene.cn/index.php?g=service&m=index&a=index&service_id=17&id=7#
28	iDNA	http://www.idna.com.cn/page/yinsixieyi.html
29	华夏基因	http://www.heal999.com/html/ETYCJC/
30	香港时代医疗集团	http://cn.medtimes.com.hk/talentgene
31	23 魔方	https://www.23mofang.com/new/protocol/service
32	中鑫基因	http://www.cntrix.com/privacySecurity.html
33	吉音树	http://www.genetree.org.cn/index.php/index.html
34	安诺优达	http://www.annoroad.com/privacy
35	华大基因检测（Genebook）	http://mall.genebook.com.cn/allBusiness/2EA0C5ED98C98598E050007F010004AF
36	新基因格医学	http://www.genegledx.com/index.php?g=Index&m=Index&a=flow
37	优迅医学	http://www.scisoon.cn/Index/bodycheck
38	元和医疗	http://www.yohecare.com/product/80.html
39	AMITA 新生健康	http://www.amitagene.com/page203.html
40	基因魔合	http://www.gene1987.com/help3.html
41	一脉基因	https://item.jd.com/41423562025.html
42	华生基因	http://hsgene.com/view-51-154.html
43	星悦康	http://www.cydjk999.com/products/tfjyjc.html
44	深大基因	http://www.shendajiyin.com/#ertong
45	淮南艾尔健康管理	http://www.aierjiankang.com/new1.asp?id=113&type1

	有限公司	=5
46	香港优质检测中心	http://www.hkyouyou.com/Page/about.html
47	安我基因	https://www.andall.com/security.htm
48	中源协和	http://www.vcanbio.com/djk.html
49	源基因	http://www.yoogene.com/index/help#privacy
50	中安基因	http://zagene.cn/index/help.html#part4
51	众安生命	https://www.zhonganbio.com/about/
52	南京壹生健康管理有限公司	http://www.lifetimejk.com/a_jkxm_jyjc/
53	裕力健康	http://www.dnaunit.com/product/278041370
54	核子基因	http://www.dnadnahezi.com/gyhz/jyjc/tfznxl/
55	美因基因	http://www.megagenomics.cn/index.php/goods/mya/id/146.html
56	FocusGene	https://www.focusgene.cn/portal/page/index/id/34.html
57	协福基因	http://www.xfjyjc.com/childgift/
58	银丰生物集团	http://www.yfswjt.com/touzi_info.php?id=47
59	博盛医疗	http://bossunhealth.com/zh/%e9%a6%96%e9%a1%b5/
60	家事录	http://www.jasloo.com/safe.php
61	知几基因	https://geneplan.com/help?tab=privacy
62	天大生物医疗	http://www.tyandar.com/product.html
63	双迪股份	http://www.dl-dd.com/product/jiyinjiance.jsp
64	华诺基因	http://www.huanuojiyin.com/col.jsp?id=103
65	人人基因	http://rrgweb.megagenomics.cn/common.jsp
66	意滋尔	http://www.easier-healthcare.com/about_syzd/ettfjyjca0d.html
67	北方基因	http://www.bjtbjx.cn/products/lm1/396.html
68	中生健康	http://www.clsh159.com/ettf.aspx

69	天和基因	https://www.thgene.cn/portal.php
70	基云惠康	https://www.genekang.com/service
71	康大基因	https://www.kangdagene.com/web_cpjs
72	云鲸基因	http://www.cloudgene.cn/products_v2.aspx
73	西安龙基因	http://www.chinalongjy.com/index.php?m=pc&a=index
74	深圳前海基因检测	http://www.jzjk365.com/product/showproduct.php?id=14
75	上海美吉医学	http://www.majordx.com/service
76	济圣基因	http://www.jshgene.com/page88
77	香港基因检测	http://www.hkgenedecode.com/tianfujiyinjiance/161.html
78	大连珍奥	http://www.zhen-ao.com/h-col-172.html
79	邃志生物	http://www.h-100.cn/index.php/default/category/31.html
80	智因互联	http://www.geneint.cn
81	杭州精皮士	http://www.geeppies.com/jzxilie1.html
82	佰美基因	http://www.lifegen.com/pre_detail.php?big_id=479&cat_id=500
83	爱康国宾	https://mall.ikang.com/product-31006.html
